# Takotsubo Cardiomyopathy following a L2–L5 Laminectomy and Fusion In Situ with Bone Morphogenic Protein

**DOI:** 10.1155/2013/724960

**Published:** 2013-03-27

**Authors:** John Weaver, Jason Eubanks

**Affiliations:** ^1^Case Western Reserve University School of Medicine, 11100 Euclid Avenue Cleveland, OH 44106, USA; ^2^Department of Orthopaedic Surgery, Division of Spine Surgery, Case Western Reserve University, School of Medicine, Cleveland, 11100 Euclid Avenue Cleveland, OH 44106, USA

## Abstract

Takotsubo cardiomyopathy (TC) is a rare, transient cardiomyopathy, with symptoms mimicking myocardial infarction. It has been reported to typically occur in postmenopausal women and is often triggered by an intense physical or emotional event with stimulation of the sympathetic response; the exact etiology, however, is uncertain. Bone morphogenic protein (BMP) is widely used in spinal fusions and has been associated with numerous perioperative complications. BMP is known to stimulate sympathetic pathways. In this paper, we present the case of a patient with a 7-hour episode of TC after a spinal fusion with bone morphogenic protein. The patient's symptoms resolved and long-term followup has been uneventful. This is the first paper to describe TC in the setting of spine or other major orthopaedic surgery and it suggests another possible area for further investigation in peri-operative events potentially associated with the use of bone morphogenic protein.

## 1. Introduction

Takotsubo cardiomyopathy (TC) is a poorly understood, rare presentation of cardiac failure in postoperative patients. TC is also called broken heart syndrome or stressed cardiomyopathy.

It presents similarly to acute myocardial infarction but has a self-limited course. Little is known about the exact etiology of TC, but it usually occurs in postmenopausal women and is often triggered by an intense physical or emotional event; however, the exact etiology is uncertain [1]. Multiple etiologies have been proposed and the connection between TC and the sympathetic response has been studied extensively. Symptoms mimic a myocardial infarction, but there is often absence of significant atherosclerotic vascular disease and patients usually make a full cardiac recovery [2]. TC is associated with a mortality rate of only 1.1% [3]. In this paper, we present the case of a patient with a 7-hour episode of TC after a spinal fusion with bone morphogenic protein (BMP). BMP is widely used in spinal fusions and has been associated with numerous perioperative complications [4]. BMP is known to stimulate sympathetic pathways [5, 6]. The following case represents the first report of TC in a patient undergoing major orthopaedic or spine surgery.

## 2. Case Report

A 75-year-old woman with a history of smoking, gastro-esophageal reflux disease, and hypertension who struggled with symptomatic neurogenic claudication secondary to severe, multilevel stenosis, and a degenerative scoliosis. She failed extensive nonoperative treatment and elected to pursue surgical intervention. She was taken to the operating room for an L1 to S1 laminectomy and L2–L5 fusion in situ with iliac crest bone graft and bone morphogenic protein (BMP). A single large, infuse sponge was employed. In this particular case, BMP was employed because of her smoking history and her multilevel pathology. Instrumentation was not used because of the relatively poor quality of her bone. She had no history or symptoms of coronary artery disease or heart failure.

Surgery was complicated by an incidental durotomy which was repaired uneventfully. The total blood loss was 550 mL. With extubation, the patient had some difficulty with congestion and was taken over to the postanesthesia care unit (PACU) on a Venturi mask. After some breathing treatments in the PACU, her airway cleared uneventfully and she was transferred to the floor.

The first two postoperative days were unremarkable. On the evening of postoperative day three, the patient developed a seven-hour course of chest pain which was described as squeezing in nature and associated with concurrent shortness of breath. AMI panels were obtained, and troponins peaked at 1.3. An ECG was obtained and demonstrated diffuse T-wave inversions and T-wave flattening. An echocardiogram was obtained and demonstrated an ejection fraction of 30%–35% with a left ventricular end-diastolic diameter of 4.6 and a left atrial diameter of 5.0.

There was akinesis of the anteroseptal wall, lateral wall, and apical wall. The echocardiogram was suggestive of an anteroseptal myocardial infarction versus a Takotsubo (stressed) cardiomyopathy. The patient subsequently underwent a left heart catheterization which demonstrated absence of atherosclerotic vascular disease. The left ventriculography demonstrated extensive anteroapical and inferior dyskinesis. The overall ejection fraction was reduced to 25%. The patient had a previously documented left ventricular ejection of 65% earlier that month.

The patient was diagnosed with TC, paroxysmal atrial fibrillation, and anxiety. The remainder of the hospital course was event-free, in particular with absence of chest pain or evidence of shortness of breath. On the day of discharge, heart rate was 72, blood pressure 108/60, respiratory rate 18, and oxygen saturation 95%. The patient was discharged to a rehabilitation center on aspirin, furosemide, lisinopril, and metoprolol for her newly diagnosed cardiomyopathy.

At the three-month orthopedic followup, the patient had no back or leg pain and was mobilizing and walking well. She was treated in a lumbosacral (LSO) brace for the first 3 months and radiographs at that time demonstrated solid bony fusion masses bilaterally from L2 to L5. At the three-month cardiac followup, the patient was doing well and did not describe any cardiac symptoms. Echocardiography showed a return of her ejection fraction to normal.

## 3. Discussion

Takotsubo cardiomyopathy is most common in postmenopausal women between the ages of 62 and 75 [7]. Relative estrogen deficiency in postmenopausal women, resulting in some endothelial dysfunction, may be one of the key predisposing factors linking them to TC [8]. Other predisposing factors include pheochromocytoma, thyroid storm, anaphylaxis, exogenous epinephrine administration, stroke, sepsis, Addison's disease, Guillain-Barre syndrome, and illicit drug use and withdrawal [9–24]. Finally, up to two-thirds of patients who experience TC carry a diagnosis of anxiety or depression [25, 26].

The relationship between stress and TC has been extensively studied. Rats pretreated with combination alpha- and beta-adrenergic receptor blockade have shown decreased instances of TC in emotional stress models [27]. Furthermore, a review of case reports found that 88 cases of TC were clearly associated with an acute or subacute emotional stressor [28]. Finally, when compared to Killip class III MI patients, TC patients had significantly higher levels of plasma catecholamines and stress neuropeptides [29].

There are multiple etiologic mechanisms that have been proposed to explain the development of TC. It has been argued that patients with an abnormal index of microvascular resistance are predisposed to TC. This is supported by reduction in coronary Doppler flow reserve and higher thrombolysis in myocardial frame counts when compared with controls [30, 31]. Another etiologic mechanism may involve left ventricular outflow tract obstruction as a result of septal bulging associated with systolic anterior motion of the mitral valve and mitral regurgitation [32]. This mechanism mimics that of hypertrophic cardiomyopathy. Multivessel epicardial spasms have also been proposed as a result of emergency coronarography showing no significant obstruction and positive provocative test in around 30% of cases [2, 33]. Finally, as previously discussed, increased catecholamines and the hormonal changes in postmenopausal women have been linked as etiologic mechanisms to the development of TC [2, 27, 29–32, 34–37].

Cardiac biomarkers are generally only slightly elevated in patients with TC. Furthermore, coronary angiography has shown that 80.6% of patients have normal coronary arteries and the remainder have luminal stenosis under 50%. Acutely all patients with TC have moderate-to-severe midventricular dysfunction and apical akinesis or dyskinesis [6].

TC has a mortality of 1.1% [6]. The majority of survivors make a full recovery in which all wall abnormalities resolve completely. Acutely ill patients with TC present with ejection fractions in the range of 20% to 49%. However, in just a matter of weeks, most patients have ejection fractions in the range of 60% to 76% [6].

Our patient was a postmenopausal 75-year-old female following a major spine surgery with significant perioperative blood loss. The physical and emotional demands of the surgery were significant for the patients, and her high anxiety was documented prior to and following her surgery. Her history of smoking and hypertension predisposed her to vascular dysfunction. Furthermore, her postsurgical anemia and challenging extubation may have also been precipitating factors in her development of TC.

Finally, it is also possible that enhanced stimulation of the sympathetic response by BMP played some role in precipitating the onset of TC. BMP has been used extensively in the last decade in spine surgery to enhance spinal fusions. Complications have been reported in association with its use, ranging from retrograde ejaculation to transient radiculitis and increased wound drainage [3]. Research has further suggested that BMP can have a profound effect on the sympathetic nervous system [4, 5]. No previous reports of cardiac events in the perioperative period have been associated to the use of BMP. It is unknown whether large doses of BMP, as was employed in this patient, could contribute to increased sympathetic tone and, consequently, cardiac events in subsets of patients.

## 4. Conclusion

TC must be included in the differential of any patient presenting with acute coronary syndrome following a stressful event, particularly if the patient is a postmenopausal woman, has a history of anxiety or depression, or has a medical condition that may predispose him/her to TC. Further inquiry into the role of BMP in potentially precipitating sympathetic mediated cardiac events might also be warranted. As the population of those undergoing major orthopaedic surgery in the United States ages, awareness of TC as a unique clinical identity in postoperative patients becomes increasingly important.
